# PRE- AND POSTOPERATIVE IMAGING METHODS IN COLORECTAL CANCER

**DOI:** 10.1590/0102-672020180001e1371

**Published:** 2018-07-02

**Authors:** Gleim Dias de SOUZA, Luciana Rodrigues Queiroz SOUZA, Ronaldo Mafia CUENCA, Vinícius Martins VILELA, Bruno Eduardo de Morais SANTOS, Felipe Souza de AGUIAR

**Affiliations:** 1 Hospital de Base do Distrito Federal; 2 Universidade Católica de Brasília, Brasília, DF, Brazil.

**Keywords:** Colorectal cancer, Colonography, Computed tomography, Magnetic resonance imaging., Câncer colorretal, Colonografia, Tomografia computadorizada, Ressonância nuclear magnética.

## Abstract

***Introduction*:**

Among the screening tests for colorectal cancer, colonoscopy is currently considered the most sensitive and specific technique. However, computed tomography colonography (CTC), magnetic resonance imaging (MRI), and transrectal ultrasonography have gained significant ground in the clinical practice of pre-treatment, screening and, more recently, post-treatment and surgical evaluation.

***Objective*:**

To demonstrate the high accuracy of CT and MRI for pre and postoperative colorectal cancer staging.

***Methods*:**

Search and analysis of articles in Pubmed, Scielo, Capes Periodicals and American College of Radiology with headings “colorectal cancer” and “colonography”. Weew selected 30 articles that contained radiological descriptions, management or statistical data related to this type of neoplasia. The criteria for radiological diagnosis were the American College of Radiology.

*****Results*** ****:**

The great majority of patients with this subgroup of neoplasia is submitted to surgical procedures with the objective of cure or relief, except those with clinical contraindication. CTC colonography is not the most commonly used technique for screening; however, it is widely used for treatment planning, assessment of the abdomen for local complications or presence of metastasis, and post-surgical evaluation. MRI colonography is an alternative diagnostic method to CT, recommended by the American Society of Gastrointestinal Endoscopy. Although there are still no major studies on the use of MRI for screening, the high resolution examination has now shown good results for the American Joint Committee on Cancer TNM classification.

***Conclusion*:**

MRI and CT represent the best means for colorectal neoplasm staging. The use of these methods as screening tools becomes beneficial to decrease complications and discomfort related to colonoscopy.

## **RESUMO**

*****Introdução*** ****:**

Dentre os testes de rastreamento de câncer colorretal, a colonoscopia é atualmente considerada a técnica de maior sensibilidade e especificidade. Entretanto, a colonografia por tomografia computadorizada (CTC), a ressonância nuclear magnética (RNM) e a ultrassonografia transrretal têm ganhado espaço significativo na prática clínica de análise pré-tratamento, rastreio e, mais recentemente, no pós-tratamento e na avaliação cirúrgica.

***Objetivo*:**

Demonstrar a alta acurácia da CT e RNM para estadiamento pré e pós-operatório do câncer colorretal.

*****Métodos*** ****:**

Busca e análise de artigos no Pubmed, Scielo, Periódicos Capes e Colégio Americano de Radiologia com descritores “câncer colorretal” e “colonografia”. Foram selecionados 30 artigos que continham descrições radiológicas, manejo ou dados estatísticos relacionados a este tipo de neoplasia. O critério de diagnóstico radiológico adotado foi o do Colégio Americano de Radiologia.

*****Resultados*** ****:**

A maioria dos pacientes portadores desse subgrupo de neoplasias é submetida a procedimentos cirúrgicos com o objetivo de cura ou alívio, exceto aqueles que possuem contraindicação clínica. A colonografia por tomografia computadorizada não é a técnica de maior utilização para rastreamento; no entanto, ela é amplamente utilizada para o planejamento de tratamento, avaliação do abdome quanto à complicações locais ou presença de metástase e avaliação pós-cirúrgica. A colonografia por RNM é método diagnóstico alternativo à CT recomendado pela American Society Gastrointestinal Endoscopy. Embora ainda não haja grandes estudos sobre o uso da RNM para rastreamento, atualmente o exame de alta resolução tem apresentado bons resultados para a classificação TNM da American Joint Committee on Cancer.

*****Conclusão*** ****:**

RNM e a TC representam os melhores meios para rastreamento de neoplasias colorretais. O uso destes métodos torna-se benéfico para diminuir as complicações e desconforto relacionadas à colonoscopia.

## INTRODUCTION

Colorectal cancer (CRC) is a multifactorial disease resulting from genetic, environmental and lifestyle factors^2^ ^,^ ^14^ ^,^ ^13^ ^,^ ^28^. It is the fifth most diagnosed cancer in Brazil, and in the Southeast occupies the second place. It is the fourth leading cause of cancer deaths in the country and almost half of the patients die in less than five years after treatment. The Mortality Information System (SIM) registered a total of 15,415 deaths as a result of this injury, with 7,387 men and 8,024 women in the year 2013^19^.

Radiological examination has relevance and evidences for preoperative staging with investigation of possible metastases (intra-abdominal, pelvic and pulmonary metastases), tumor infiltration or extension, and postoperative evaluations together with anatomopathological staging^1^ ^,^ ^10^ ^,^ ^11^ ^,^ ^15^ ^,^ ^17^ ^,^ ^25^ ^,^ ^26^ ^,^ ^28^.

The objective of this study was to demonstrate the high accuracy of computed tomography (CT) and magnetic resonance imaging (MRI) for pre and postoperative colorectal cancer staging.

## METHODS

The methodology used was to search for and analyze articles in Pubmed, Scielo and Periódicos Capes, besides the American College of Radiology with descriptors of “colorectal cancer” and “colonography”. Were selected 30 articles that contained radiological descriptions, management or statistical data related to this type of neoplasia. Additional statistical data were obtained from the Datasus system, the Mortality Information System (SIM), the National Cancer Institute (INCA) and the Demographic Census of 2010. The criteria for radiological diagnosis were the American College of Radiology.

## RESULTS

### Diagnosis

It is essential to detect the CRC in the initial stages of injury evolution, in order to reduce morbidity and mortality. For this reason, in the suspicion of the clinical history and the physical examination, it is mandatory to perform a proctological examination (rectal examination)^3^ ^,^ ^9^ ^,^ ^15^.

The “Projeto Diretrizes” recommends that the identification of the site of the lesion can be done by retosigmoidoscopy. However, colonoscopy has the advantage of identifying small lesions and providing histopathological material, so it is the preferential examination at diagnosis. During colonoscopy, if polyps are found outside the resection area of the main lesion, they can be removed at this moment^15^.

Contrast radiological examination of the colon (opaque enema) should be reserved for when there is no access to the colonoscopy or when there is any contraindication to this examination^3^ ^,^ ^16^ ^,^ ^23^.

However, CT colonography (CTC) has been proposed as a viable alternative, due to the greater acceptance of patients with colonoscopy-like efficacy and advantages related to the speed of the examination, less invasion, no need for sedation and allows the patient to return to their activities soon after performing the procedure^3^ ^,^ ^22^.

### Screening

The main way to identify CRC in early stages of evolution is through proper screening. Colonoscopy has been, together with the search of fecal occult blood and carcinoembryonic antigen, the main screening tools^3^ ^,^ ^6^ ^,^ ^10^ ^,^ ^30^.

The reduction in mortality due to fecal occult blood tests in patients older than 50 years represents about 15-33% of the reduction in mortality, just as the colonoscopy with removal of polyps for anatomopathological analysis reached 53% in this reduction^2^ ^,^ ^7^ ^,^ ^15^ ^,^ ^28^.

The current guideline determines that low-risk individuals, aged 50 and older, are required to conduct annual fecal occult blood tests and rectosigmoidoscopy every five years. From the age of 60, perform colonoscopy or opaque enema every 10 years. Patients exposed to risk factors should begin screening at age 40, including colonoscopy^2^ ^,^ ^11^ ^,^ ^15^.

The Amsterdam II criteria define genetic testing for individuals with a history of hereditary nonpolyposis colorectal cancer in the family when: three or more relatives had colon cancer (or other cancer associated with hereditary nonpolyposis colorectal, like uterus cancer, small intestine, urethral or pelvic kidney) and at least one of them is a first degree relative; two or more generations of the family have colon cancer; one or more relatives were diagnosed with colon cancer before age 50. Screening should be started around the age of 21 in affected patients and subsequently be performed at least every five years. The Bethesda criteria modify those of Amsterdam II to include, in the evaluation, the patients who had relatives with colonic adenomatous polyps, in addition to the CRC^2^ ^,^ ^10^ ^,^ ^14^.

### Screening by radiological tests

Most of the guidelines endorsed by the World Health Organization divide CRC screening tools into two main categories: those capable of detecting both adenomatous polyps and cancer (sigmoidoscopy, barium enema, MRI colonography, CTC and colonoscopy), and those screening (fecal occult blood test, immunohistochemical stool test and fecal DNA test)^3^ ^,^ ^5^ ^,^ ^9^ ^,^ ^15^ ^,^ ^23^.

CTC (also known as “virtual colonoscopy”) was introduced in 1994 as a less invasive method of colon analysis using helical CT. An assay performed on 307 asymptomatic subjects using CTC with a computerized tomograph of 64 detectors demonstrated sensitivity and specificity of 91% and 93%, respectively, for polyps greater than 6 mm and 92% and 98%, respectively, for polyps greater than or equal to 10 mm^2,^ ^3^.

A cross-sectional study on patient preference in CRC screening comparing CTC with colonoscopy, published in the Brazilian Journal of Radiology, showed that 86% reported preferring CTC^22^.

Thus, CTC can be considered as an alternative to diagnostic colonoscopy, with advantages related to its convenience and patient acceptance.

### Staging methods

For CRC staging is necessary to segment the evaluation times into: pre, intra and postoperative.

#### *Preoperative staging*

The main objective is to identify the local and regional extension of the primary lesion; however, it is necessary to search its extension to other locations. The dosage of carcinoembryonic antigen (CEA) is relevant in the prognosis^9^ ^,^ ^23^.

Investigation of intra-abdominal and pelvic metastases should be performed by ultrasonographic or CT examination. Investigations of pulmonary metastases can be investigated from the clinical parameter with chest X-ray or CT scan^15^ ^,^ ^16^ ^,^ ^30^.

The initial staging of the CRC has been widely performed from the clinic and the imaging tests: CT, MRI and transrectal ultrasound. This methodology represents clinical staging, which is necessary for therapeutic evaluation, definition of surgical margins for healing intent and precise locations for surgical staging^3^ ^,^ ^16^.

Nuclear medicine methods also represent an alternative to preoperative staging.

#### *Computed tomography*

Initially CT was the first imaging test used for preoperative staging and initial studies demonstrated accuracy of 85-95% of the exam. However, controlled studies showed an accuracy of 50-70% depending on the stage of the neoplasia. CT is still recommended in the initial evaluation of all patients scheduled for CRC because of their ability to obtain a rapid global assessment and a low number of complication^3^ ^,^ ^6^ ^,^ ^16^.

There is a variation of accuracy depending on the location of the lesions, with T2 and T3 being better accessed than T4 lesions. Another relevant factor is the difficulty in determining the penetration of the wall tissue (“T” stage) by CT. The finding of perirectal spicules may be a confounding factor with tumor desmoplastic inflammation^3^ ^,^ ^16^. The specificity for lymph node determination may reach 45%. The detection of distant metastases has good sensitivity and specificity, ranging from 85-97%^16^.

CTC proved to be a valid instrument both in primary identification and in extracolonic metastasis. It is beneficial for incomplete colonoscopy, with an accuracy of 81%, sensitivity of 93% and specificity of 97% for the detection of polyps greater than 1 cm, with sensitivity and specificity being 86% for polyps smaller than 1 cm^3,^ ^23^ ^,^ ^30^.

#### *Magnetic resonance*

Colonography by MRI is an alternative diagnostic method to CT, recommended by the American Society of Gastrointestinal Endoscopy. Studies have shown that it has an accuracy of 58% for the detection and local staging of rectal cancer, with the same precision of CT, despite the complications related to radiation^18^.

Polyps and adenomas smaller than 6 mm have less clinical relevance, which increases the importance of studying MRI as a screening method for colon cancer, since the sensitivity for large polyps larger than 10 mm is 84%^18^.

Most of the research to date has been done with 1.5 Tesla MRI. The current hypothesis is that the 3 Tesla examination would substantially increase image quality. Hüneburg et al.^18^ compared MRI with 3T vs. colonoscopy. The results shown corroborate the idea that MRI has low accuracy to identify small polyps. However, in relation to lymphatic metastases, it was similar to CT with sensitivity of 85%. MRI was slightly superior for the detection of hepatic metastases^3^ ^,^ ^6^ ^,^ ^16^ ^,^ ^18^. Despite, the use of endorectal coils showed results with improvement to determine the penetration of the wall tissue. But there is no consensus on the routine use of endorectal coils in clinical practice. The main limitations of the apparatus are: determination of suprarenal, pelvic lateral and mesenteric lymph nodes; limitation of imaging in obese patients^3^ ^,^ ^16^.

The diffusion-weighted MRI showed better sensitivity and accuracy when compared to the traditional gadolinium contrasted test. It does not use contrast, is more sensitive than CT in detecting metastases and has a greater potential for the evaluation of preoperative TNM staging and postoperative follow-up of CRC ^3^ ^,^ ^16^ ^,^ ^23^ ^,^ ^30^.

#### *Transrectal ultrasonography*

It is cited by the ACR as a standard test for preoperative CCR staging because of its ability to detect the level of penetration and the distinction between the layers of the intestinal wall. The accuracy to determine the T stage may reach 84.6%. However, overestimation of the stage may be a problem, especially in T2 and T3 level injuries. The detection of lymph node involvement is difficult, although greater than CT; its sensitivity is low (50-57%). Therefore, lymph nodes affected by micrometastasis in the early stages of CRC may be one of the great factors of recurrence, especially in the pelvic area^3^ ^,^ ^6^ ^,^ ^15^ ^,^ ^16^.

#### *Image staging*

**FIGURA 1:**
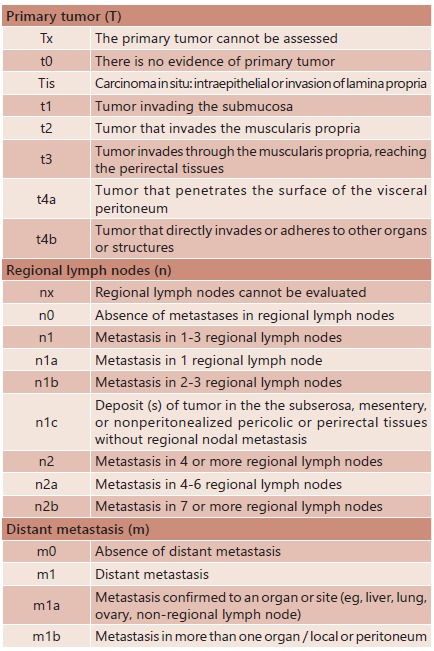
TNM system of the American Joint Committee on Cancer (AJCC)^7^

Staging is the major component of the surgical predictor. Method is based on the American Joint Committee on Cancer (AJCC) TNM system (Figure 1) and replaces the previous Duke and Astler-Collier systems.

CRC staging depends on the depth of the wall invasion and its accuracy is critical for defining the treatment and prognosis of patients. The difference between the treatment of colon and rectum cancer indicates a different evaluation of the imaging methods^3^ ^,^ ^5^ ^,^ ^13^ ^,^ ^14^.

#### *Staging of colon cancer*

The most indicated imaging method is CT with contrast, previously used only for detection of metastases. There is the predilection for images after distension of the colon with iodinated contrast, water or air for better visualization of the lesion.

Good visualization is performed by the analysis of images in the hepatic portal contrast and equilibrium phase, which allows the identification of primary lesions and liver metastases. The arterial phase, although suppressed in some services, may be useful for the delimitation of the primary tumor^3^ ^,^ ^5^ ^,^ ^13^ ^,^ ^16^. The interpretation should provide the location of the lesion, relationships with adjacent structures, possibility of invasion of another organ and relation with the retroperitoneal fascia^3^ ^,^ ^5^.

By analyzing the T stage, it is known that CT is not able to easily differentiate the transition between mucosa and submucosa; therefore, it is not possible to differentiate T1/T2 tumors. However, the accuracy of the differentiation between T1/T2, T3 and T4 is 80%^3^ ^,^ ^5^ ^,^ ^16^.

T1/T2 tumors present as vegetative lesions or asymmetric focal thickenings of the colonic wall, with smooth external contours and without intensification of mesocolic adipose tissue.

T3 tumors tend to show protrusion or bulging of the contours of the external surface of the intestinal wall, irregularities in its contour or frank signs of direct tumor extension with pericolic fat infiltration^16^.

Tumors of T4 classification infiltrate the visceral peritoneum of adjacent organs and maintain an intimate relationship with other organs.

The tomographic criteria used to indicate preoperative chemotherapy are: T3 tumors with extramural extension greater than 5 mm and T4 tumors that penetrate the visceral peritoneum or that affects adjacent organs^3^ ^,^ ^5^ ^,^ ^13^ ^,^ ^16^.

The identification of affected lymph nodes is performed by analysis of the pathological dimensions (diameter greater than 1 cm in mesentery, retroperitoneum, internal hilar and inguinal chains and greater than 0.5 cm in the mesorectum), clustered or irregular lymph nodes. The method is limited in the identification of micrometastases^3^ ^,^ ^5^ ^,^ ^11^ ^,^ ^30^.

The search for metastases mainly occurs in the liver. Hepatic lesions often present as focalized and hypovascularized, more evident in the portal contrast phase.

#### *Staging of rectal cancer*

**FIGURE 2:**
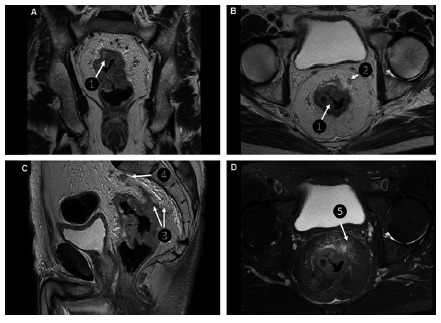
T2-weighted coronal (A) and axial (B) sequences demonstrating vegetative lesion on the retosigmoid compromising all layers of its wall (A and B - ❶ and arrow), and with small satellite lymph node (B - ❷ and arrow) . Sagittal T2 - weighted sequences (C) and axial T2FSE (D) characterizing the extension of lesion by infiltration into posterior pararectal fat (C - ❸ and arrows) and anterior pararectal fat bordering the tumor lesion perceived as hypersignal (D - ❺ and arrow ). Parasacral satellite lymph node (C - ❹ and arrow).

MRI is the modality of choice for staging of rectal cancer. Using this imaging examination, it is possible to demonstrate the relation of the tumor with the adjacent structures and the wall of the intestine (Figures 2A and B). The muscular layers of the mucosa, submucosa, and muscularis propria can be identified, as well as the perirectal fat and the mesorectal fascia (Figure 2C) ^20^ ^,^ ^29^.

Non-mucinous tumors appear as intermediate signal areas in the T2 sequences and with restriction at diffusion. The mucinous ones present with high intensity (similar to liquid). There may be several aspects: polypoid, ulcerative, semi-circumferential or circumferential. 

Currently high resolution MRI has shown good results for the AJCC TNM classification^3^ ^,^ ^5^ ^,^ ^11^ ^,^ ^14^ ^,^ ^30^.

The T stage is characterized by the invasion of the primary tumor through the rectal wall and its relation with its own submucosa and muscular propria (Figure 2C).

Tumors in the T1 stage are represented in MRI as areas of abnormal intermediate signal intensity replacing the submucosal hypersignal.

Those classified as T2 reach the muscularis mucosae, but without extension to the mesorectal adipose tissue.

**FIGURE 3:**
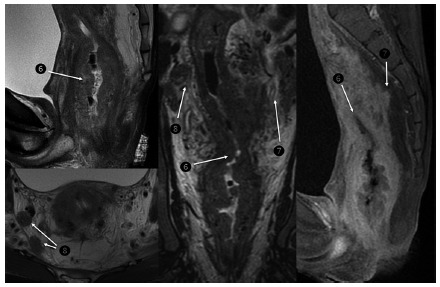
Sagittal T2 sequences (A), T2 (B) coronal reconstructions, sagittal (C) and axial T2 (D) demonstrating a vegetative lesion that reduces its lumen (A, B and C - ❻ and arrows) compromising all the layers of its wall. Infiltration of the pararectal fat by contiguity (B and C - ❼ and arrows). Deep inguinal lymph nodes involvement (B and D - ❽ and arrows).

Tumors in the T3 stage exceed the muscularis propria and reach mesorectal fat, characterized by areas of abnormal nodular intermediate intensity present in the mesorectal fat (Figures 3B and 3C) ^3,5,14,16,23^.

Tumors in stage T4 are characterized by invasion of adjacent organs and structures or by perforation of peritoneal deflection.

Lymph nodes with homogeneous and uniform signal are not considered suspicious. Lymph nodes with irregular borders, signs of different intensity and increased in size are considered suspicious lymph nodes (Figures 3B and D). From one to three affected lymph nodes, the classification is N1; if four or more N2^29^.

## CONCLUSION

Imaging tests are essential for CRC staging and diagnosis. CT and MRI should be widely used, thus representing less complication and discomfort in relation to colonoscopy. CT is consolidated for staging of colorectal neoplasms; however, MRI represents a gain because there is no exposure to radiation. Although there are some limitations regarding the detection of small polyps, these imaging tests should be used to screen CRC for identifying potentially neoplastic lesions in a non-invasive way.
